# Biomechanical evaluation of the triangular support structure of the proximal femoral bionic nail compared to conventional long intramedullary nails for subtrochanteric fractures

**DOI:** 10.3389/fbioe.2025.1579842

**Published:** 2025-05-30

**Authors:** Yanjiang Yang, Dongwei Wu, Xiaodong Cheng, Wei He, Wei Chen, Yingze Zhang, Qi Zhang

**Affiliations:** ^1^ Trauma Emergency Center, Third Hospital of Hebei Medical University, Shijiazhuang, Hebei, China; ^2^ Orthopaedic Research Institute of Hebei Province, Shijiazhuang, Hebei, China; ^3^ Key Laboratory of Biomechanics of Hebei Province, Shijiazhuang, Hebei, China; ^4^ Hebei Chest Hospital, Shijiazhuang, Hebei, China; ^5^ NHC Key Laboratory of Intelligent Orthopaedic Equipment, Shijiazhuang, Hebei, China; ^6^ Hebei Orthopaedic Clinical Research Center, Shijiazhuang, Hebei, China

**Keywords:** biomechanics, finite element analysis, subtrochanteric fracture, proximal femoral bionic nail, triangular support structure

## Abstract

**Purpose:**

The aim of this study was to compare the biomechanical results of long proximal femoral bionic nail (PFBN) and three conventional intramedullary nails in the treatment of subtrochanteric fractures (STFs).

**Methods:**

Using finite element analysis, we compared the therapeutic efficacy of four long intramedullary nails: the PFBN, reconstruction nail (RCN), InterTAN nail (ITN), and proximal femoral nail antirotation (PFNA) for the treatment of Seinsheimer type IIIA and type V STFs. The biomechanical stability of the implants was evaluated by calculating of von Mises stress (VMS), contact pressure and displacement for three loading scenarios.

**Results:**

The results showed that the PFBN group had the lowest VMS values under axial, bending and torsional loads. Under axial loading conditions, the VMS of PFBN was 480.04 MPa, followed by ITN (726.39 MPa), PFNA (730.48 MPa), and RCN (837.24 MPa) in the type V fracture groups. In the PFBN group, the contact pressure was 19.22 MPa and the tangential micromotion was 0.089 mm for the type IIIA group, 23.69 MPa and 0.08 mm for the type V group. Compared to the ITN, PFNA and RCN groups, the PFBN group exhibited the lowest contact pressure and tangential micromotion at the fracture sites.

**Conclusion:**

The superior biomechanical properties of the PFBN under axial, bending, and torsional loads not only reduced stress at the fracture site, but also improved structural stability.

## Introduction

Subtrochanteric fractures (STF) are a challenging subset of proximal femoral fractures, accounting for approximately 10%–30% of all hip fractures in all different age groups (Zhang et al., 2022b; Arshi et al., 2023; Grønhaug et al., 2023). STFs occur approximately 5 cm below the lesser trochanter to the distal end of the femoral shaft, and can be the result of either high-energy trauma in the young or osteoporosis in the elderly. Effective treatment of these fractures is critical due to the complex biomechanical environment of the proximal femur, including severe axial, rotational, and lateral instability.

Conventional intramedullary nails (IMNs), such as the Reconstruction nail (RCN), InterTAN nail (ITN), and Proximal Femoral Nail Antirotation (PFNA), are widely used for internal fixation of STF. However, the dual parallel screw design, interlocking dual screw design, and helical blade design have all failed to resolve the clinical challenge of high mechanical complications in STF. Specifically, the incidence of screw breakage, Z effect, cutting, and bone nonunion ranges from 7% to 20%, with more than 50% of these cases requiring revision surgery (Kwak et al., 2021; Zhang et al., 2024).

In recent years, the advancement of biomechanics has led researchers to apply bionic fixation concepts that mimic the natural structure and function of bone to clinical challenges (Yang et al., 2024a; Zhang et al., 2024). The triangular support structure of the proximal femoral bionic nail (PFBN) simulates the trabecular structure of the proximal femur, and its mechanical conduction path closely resembles the natural state of the femur (Zhang et al., 2024). Clinical data showed that screw-related complications were improved when PFBN was used to treat proximal femoral fractures. However, there is still a lack of biomechanical data evaluation for long PFBN in Seinsheimer type IIIA and type V STFs. In this study, we aimed to compare the biomechanical data of four types of long IMNs, PFBN, RCN, PFNA, and ITN, for the treatment of STF.

## Materials and methods

Ethical approval for this study (NO. KS2022-011-1) was provided by the Medical Ethics Committee of the Hebei Medical University Third Hospital. These methods were carried out in accordance with the guidelines of Helsinki Declaration.

### Finite element model establishment

**FIGURE 1:**
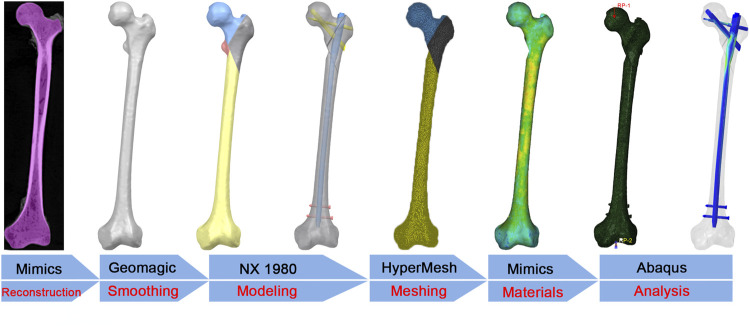
The flow chart of the finite element analysis.

As shown in Figure 1, the flow chart illustrated the finite element analysis process. A male femur specimen stored at −80°C was selected as the model object. Prior to further analysis, a radiographic examination was performed to exclude bone abnormalities such as severe osteoporosis, deformities, and tumors. The femur was scanned by computed tomography (SOMATOM Definition AS scanner, Siemens, Germany, thickness, 0.625 mm; resolution, 512 × 512 pixels). The DICOM format data were imported into Mimics 20.0 software (Materialise, Leuven, Belgium) for geometric model reconstruction and exported in stereolithography (STL) format. The STL file was imported into Geomagic Studio 13.0 software (Geomagic Company, United States), and solidification was achieved by operations such as denoising, smoothing, and surface fitting.

### Establishing implants and assembled model

Four models (PFBN, PFNA, InterTAN, and RCN) were designed using NX 1980 software (Siemens Company, United States). All IMNs had the same diameter (10 mm), angle (125°), and length (400 mm). The STFs were designed according to the Seinsheimer classification: Type IIIA (spiral with lesser trochanteric portion of the third fragment) and Type V (subtrochanteric-intertrochanteric fracture). The IMNs were assembled with the fracture models to ensure consistent positioning within the femoral head and shaft. The solid models were meshed with C3D4 elements and checked for quality using HyperMesh Desktop 14.0 (Altair Company, United States).

### Material properties and boundary conditions

Bone density correlates with material properties. Therefore, the material properties of each femoral model were based on the Hounsfield units from the CT scan data in Mimics 20.0 software. The mathematical formulas are as follows (Equation 1) (Yang et al., 2024a), where *ρ* was the bone density, Hu represented the Hounsfield units, *E* was Young’s modulus, and *ν* was Poisson’s ratio. The models were imported into Abaqus 6.14 software (Dassault Company, United States).

$$\begin{array}{c} \;\rho \left(g/{\text{cm}}^{3}\right)=0.000968\times Hu+0.5\\ \text{ If }\rho \leq 1.2g/{\text{cm}}^{3};E=2014\times \rho^{2.5}\left(MPa\right),\nu =0.2\\ \text{ If }\rho >1.2g/{\text{cm}}^{3};E=1763\times \rho^{3.2}\left(\text{MPa}\right),\nu =0.32 \end{array}$$ (1)

All the implants were assigned as titanium alloy (Ti-6Al-4V), with *E* of 110 GPa and Poisson’s ratio (*ν*) of 0.3. All contact types were defined according to the Coulomb friction law: bone–bone (friction coefficient: *μ* = 0.46), bone–implant (*μ* = 0.3), and implant–implant (*μ* = 0.2) (Yang et al., 2024b). The screw thread was tied to the bone.

**FIGURE 2:**
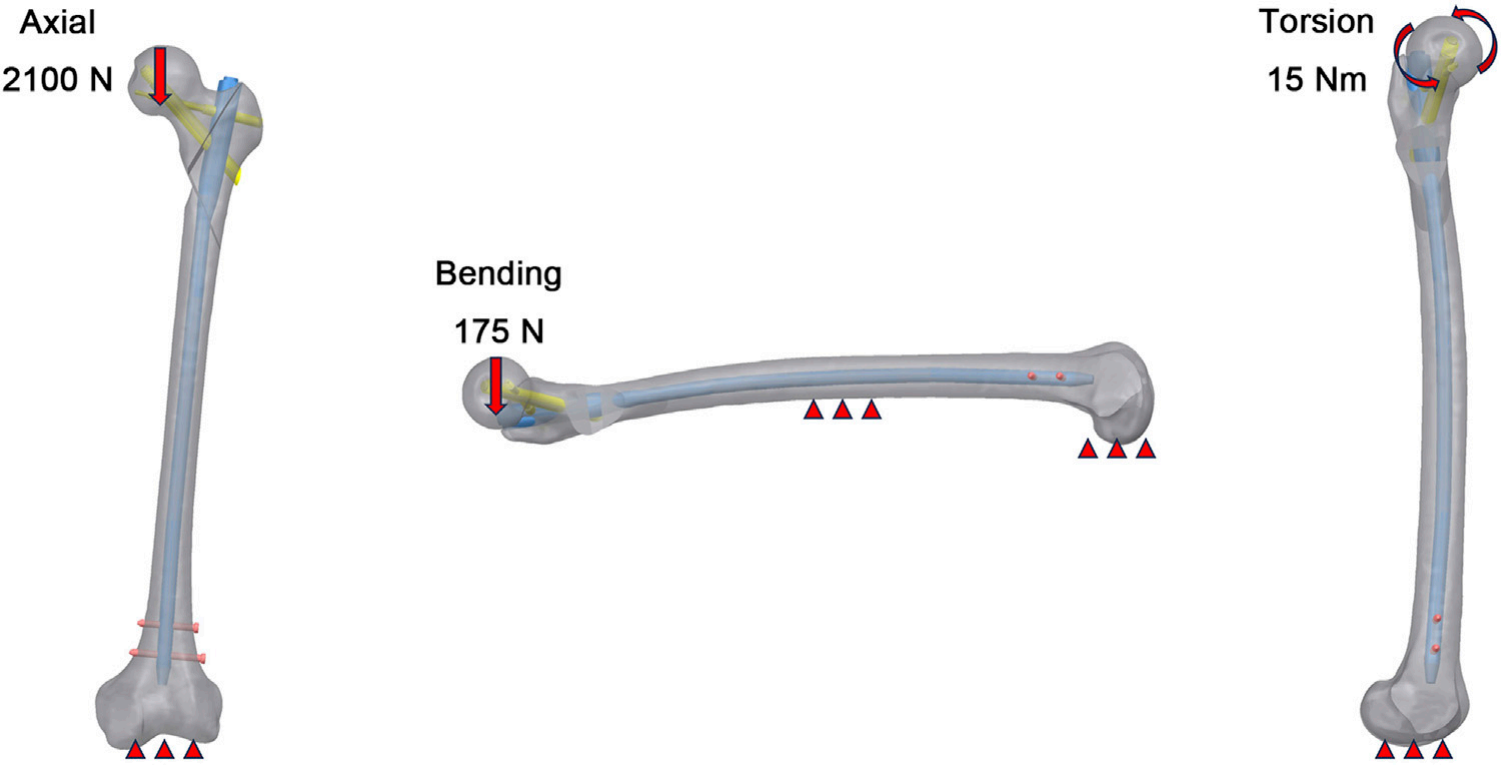
Boundary conditions for axial, bending, and torsional loads.

As shown in Figure 2, three load conditions were simulated, including axial, bending, and torsional loads. The distal end of femur was completely fixed by constraining 6 degrees of freedom. In the axial load case, a uniform load equivalent to 300% of a 70 kg body weight was applied to the femoral head to simulate the maximum load experienced by the hip joint during walking. The 175 N lateral load was selected to simulate forces on the femur during activities like lateral bending in normal ambulation. For the boundary of the torsional load case, a 15 Nm torsional load was applied on the surface of femoral head along the axis of the femoral neck and representing the maximum load applied to the femoral head during normal human gait.

### Verification of finite element models

Mesh convergence tests were performed in the previous study (Yang et al., 2024b). The mesh size was set to 2.0 mm. The implant components were meshed with 1.0 mm, which was fine enough to preserve geometric features. The node and element numbers of the four groups were shown in Table 1.

**TABLE 1:** Amounts of nodes and elements of four components.

Components	Nodes	Elements
PFBN	131655	598076
InterTAN	130127	586328
PFNA	138909	641935
RCN	133495	613970

In our study, the femoral model was biomechanically validated using the BOSE ElectroForce 3520-AT (BOSE Company, United States) and the high-speed camera integrated within the GOM non-contact optical strain measurement system (GOM GmbH, Germany). Under the same loading and boundary conditions as the biomechanical experiment, the displacement values at the corresponding position were calculated for the normal femur finite element model. The high consistency between experimental measurements and simulation results further validated the stability of the model over multiple runs. The result indicated that our model was appropriate for the subsequent study.

### Evaluation indices

Von Mises stress (VMS) and displacement of the four models were calculated to evaluate the biomechanical stability under axial, bending and torsional loads. The stability of the fracture surface was assessed through tangential micromotion and contact pressure under axial load. CSLIP1 and CSLIP2 refer to the Component SLIP (Sliding Micromotion) values in two orthogonal directions at the interface of two contacting surfaces (Zhang et al., 2023). These values represent the tangential micromotions (sliding or relative motion) that occur between the surfaces due to external forces or displacements. Therefore, d was used to characterize the tangential micromotions of the fracture surfaces of the STF.

$$d=\sqrt{{\text{CSLIP}1}^{2}+{\text{CSLIP}2}^{2}}$$

## Results

### The VMS distributions of the implant models under axial, bending and torsional loads

**FIGURE 3:**
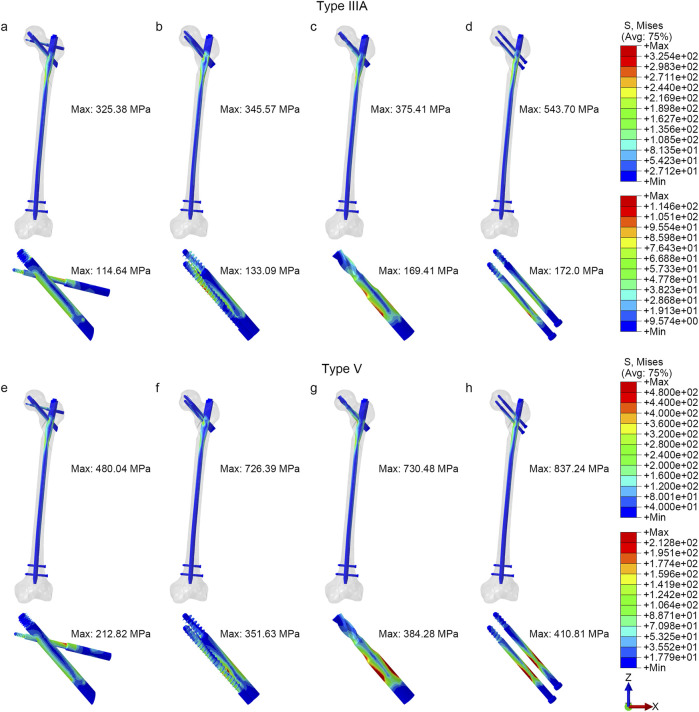
The von Mises stress distribution of implants **(a, e)** PFBN, **(b, f)** InterTAN, **(c, g)** PFNA, **(d, h)** RCN.

**FIGURE 4:**
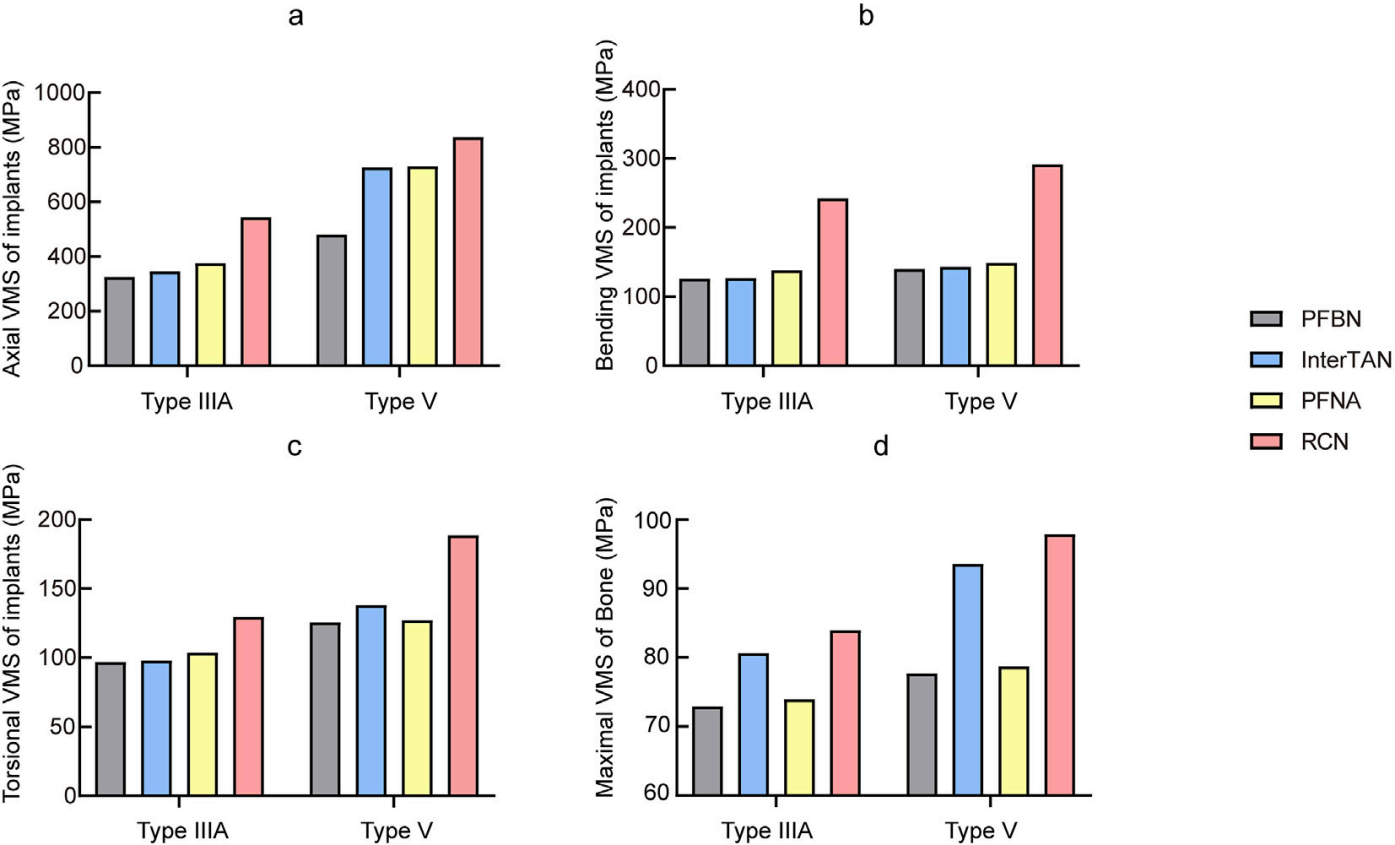
Comparison of von Mises stress of all fracture models with different implants **(a)** Axial, **(b)** Bending, **(c)** Torsional, **(d)** Bone.

Under axial loading, PFBN showed the lowest implant stress among the four IMN types (Figures 3, 4a). The PFBN stress value was 325.38 MPa in type IIIA STF and 480.04 MPa in type V fractures. On the contrary, the RCN showed the highest stress value, especially in type V fractures (837.24 MPa). Compared to InterTAN, PFNA, and RCN, PFBN decreased by approximately 5.84%, 13.33%, and 40.15% in type IIIA fractures and 33.91%, 34.29%, and 42.73% in type V fractures, respectively. The internal fixation screws of femoral head were analyzed separately. The cross-design stress distribution of the fixation and support screws (212.82 MPa) was superior to that of the double parallel screws (410.81 MPa), interlocking double screws (351.63 MPa), and helical blade (384.28 MPa).

Under bending load conditions (Figure 4b), the PFBN exhibited relatively low stresses compared with other intramedullary nails, 125.95 MPa for type IIIA fractures and 139.74 MPa for type V fractures. For type V fractures, for example, the PFBN showed a reduction of 2.48%, 6.06%, and 52.15% when compared with the InterTAN, PFNA, and RCN, respectively.

For torsional loading (Figure 4c), the PFBN also maintained lower stresses, 96.87 MPa for type IIIA fractures and 125.50 MPa for type V fractures. The PFBN was reduced by approximately 1.02%, 6.41%, and 25.18% for type IIIA fractures and 8.99%, 1.25%, and 33.43% for type V fractures, respectively, when compared with InterTAN, PFNA, and RCN.

### The VMS distributions of femur under axial loading

Under axial loading (Figure 4d), the femoral VMS was 72.89 MPa in the PFBN group, 80.63 MPa in the InterTAN group, 73.90 MPa in the PFNA group, and 83.95 MPa in the RCN group for type IIIA STF. The stability of type V STF was reduced and the corresponding femoral VMS were increased: 77.67 MPa in the PFBN group, 93.54 MPa in the InterTAN group, 78.68 MPa in the PFNA group, and 97.85 MPa in the RCN group.

### The displacement distributions of the models

**FIGURE 5:**
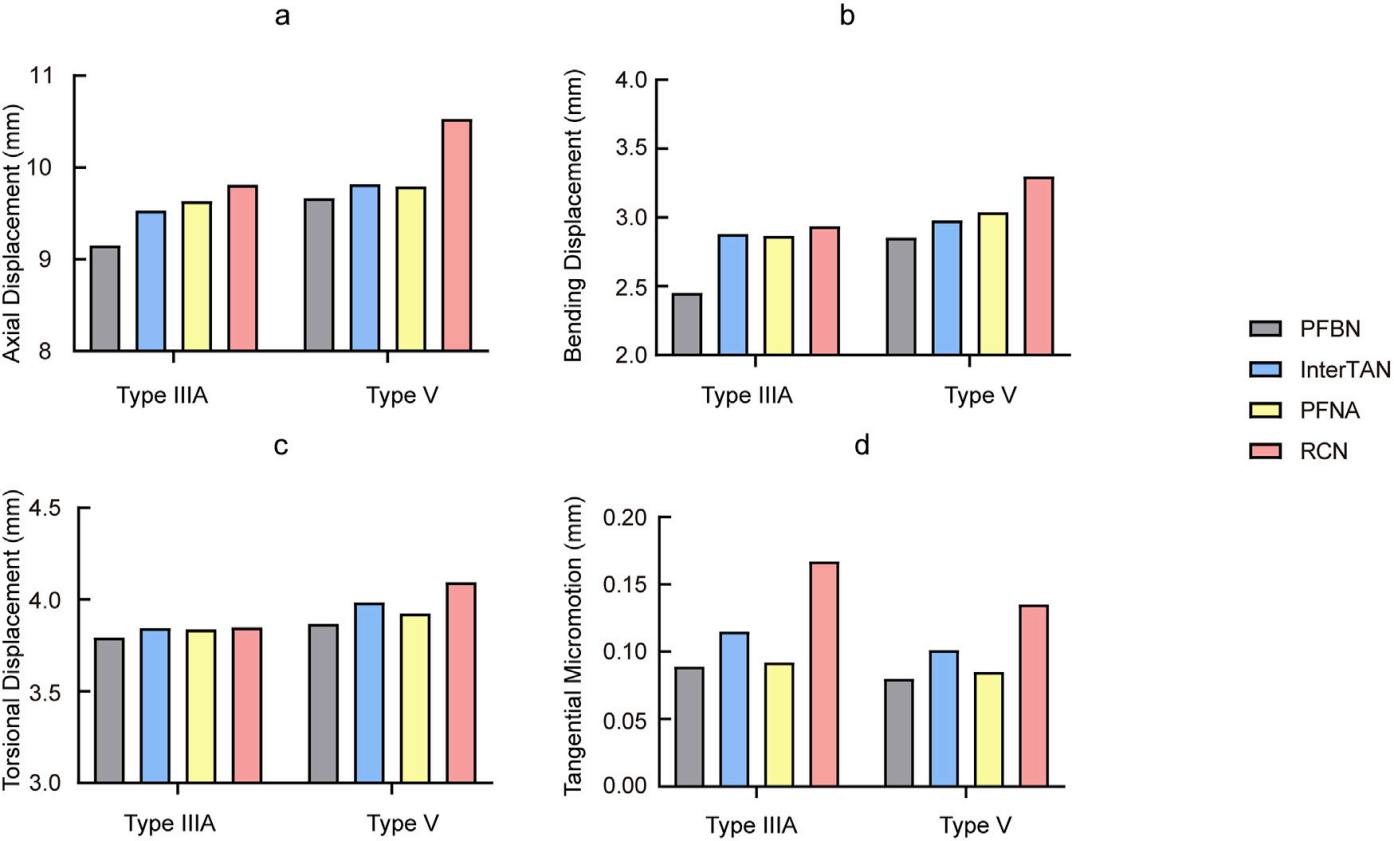
Comparison of displacement and tangential micromotion of all fracture models with different implants **(a)** Axial, **(b)** Bending, **(c)** Torsional, **(d)** Tangential micromotion.

Under axial loading (Figure 5a), the whole displacement of the PFBN group for type IIIA STF was 9.150 mm, 9.529 mm for InterTAN, 9.632 mm for PFNA, and 9.812 mm for RCN. The whole displacement of the PFBN group for type V STF was 9.665 mm, 9.816 for InterTAN, 9.792 mm for PFNA, and 10.530 mm for RCN. Under bending load (Figure 5b), the PFBN group also showed more stability, with displacement of 2.453 mm and 2.854 mm for type IIIA and type V fracture models, respectively. Under torsional loading (Figure 5c), the difference in displacement between the groups was relatively minor. The displacement of the type IIIA and type V fracture models in the PFBN group was 3.793 mm and 3.867 mm, respectively.

### Contact pressure and tangential micromotion of fractured surface

**FIGURE 6:**
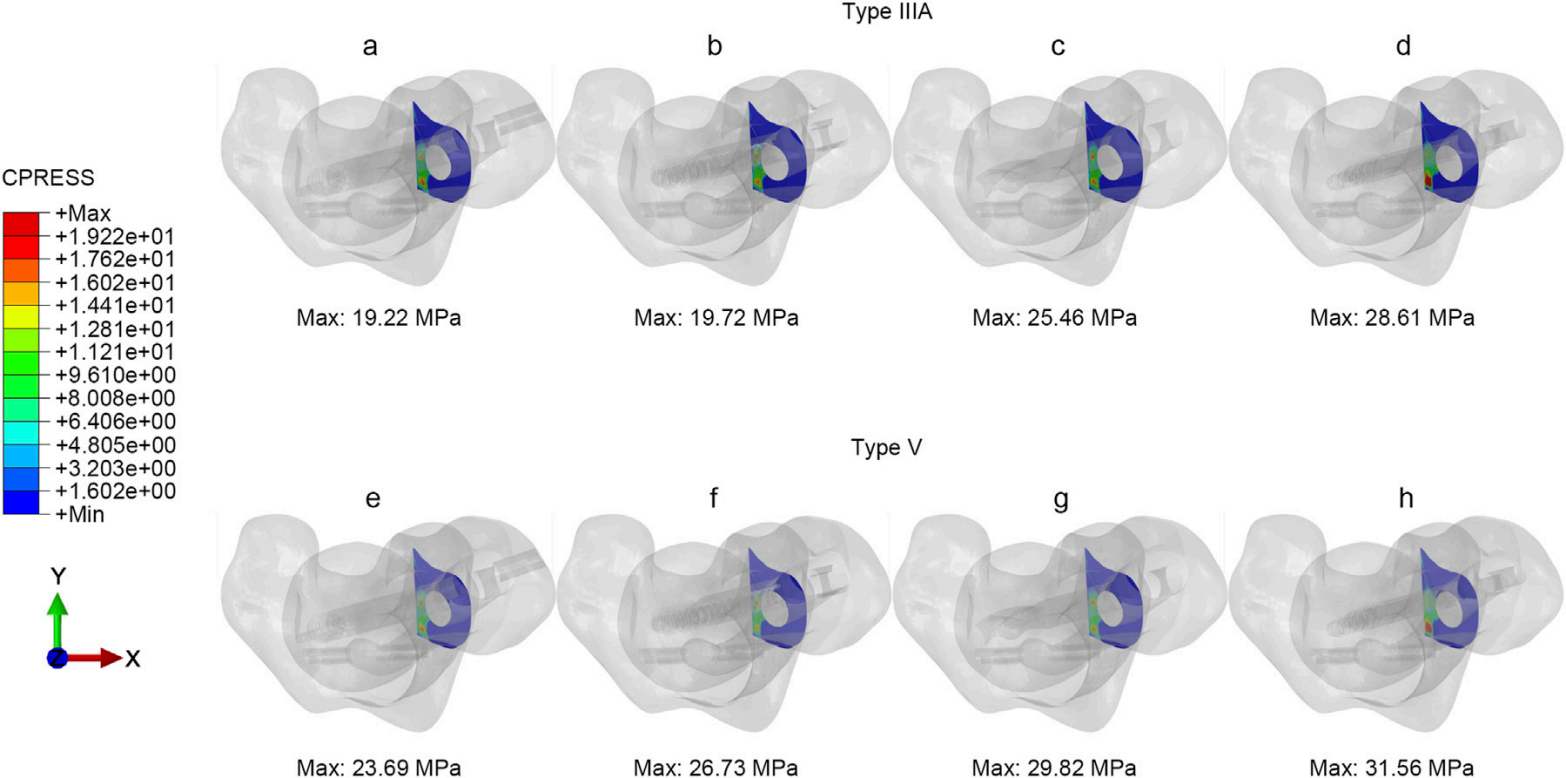
The contact pressure of fracture interfaces under axial load **(a, e)** PFBN, **(b, f)** InterTAN, **(c, g)** PFNA, **(d, h)** RCN.

Under axial loading conditions, the contact pressure and tangential micromotion of the fracture interface can reflect the stability of the fracture sites. Figures 5d, 6 illustrated the micromotion and contact pressure of the fracture surfaces in each group model. In the PFBN group across the four models, the contact pressure was 19.22 MPa and the tangential micromotion was 0.089 mm for the type IIIA group, 23.69 MPa and 0.08 mm for the type V group. Compared to the InterTAN, PFNA and RCN groups, the PFBN group exhibited the lowest contact pressure and tangential micromotion at the fracture sites, indicating its superior stability. This stability is beneficial for osteoporotic patients as it could help mitigate postoperative mechanical complications, such as screw cut-out, varus collapse.

The biomechanical evaluation parameters of the four intramedullary nails under axial, bending and torsional loading were shown in Table 2.

**TABLE 2:** The biomechanical evaluation of four intramedullary nails for three loading scenarios.

Load	Parameters	PFBN		InterTAN		PFNA		RCN	
		Type IIIA	Type V	Type IIIA	Type V	Type IIIA	Type V	Type IIIA	Type V
Axial	VMS of implant models (MPa)	325.38	480.04	345.57	726.39	375.41	730.48	543.70	837.24
	VMS of femur models (MPa)	72.89	77.67	80.63	93.54	73.90	78.68	83.95	97.85
	Displacement (mm)	9.150	9.665	9.529	9.816	9.632	9.792	9.812	10.530
	Contact pressure (MPa)	19.22	23.69	19.72	26.73	25.46	29.82	28.61	31.56
	Tangential micromotion (mm)	0.089	0.080	0.115	0.101	0.092	0.085	0.167	0.135
Bending	VMS of implant models (MPa)	125.95	139.74	126.89	143.29	138.00	148.76	242.00	291.63
	Displacement (mm)	2.453	2.854	2.880	2.977	2.865	3.038	2.936	3.297
Torsional	VMS of implant models (MPa)	96.87	125.50	97.87	137.89	103.50	127.09	129.56	188.53
	Displacement (mm)	3.793	3.867	3.844	3.983	3.837	3.925	3.848	4.095

## Discussion

Our finite element analysis demonstrated that the PFBN exhibits superior biomechanical performance compared to conventional IMNs. Specifically, the PFBN showed a 42.73% reduction in maximum VMS at the fracture site under axial loading, a 52.15% improvement in bending stability, and a 33.43% enhancement in torsional strength. These results suggest that the PFBN effectively distributes mechanical loads more evenly, thereby reducing the likelihood of complications such as Z effect, cut-out, and varus collapse.

Seinsheimer type V fractures, which are subtrochanteric fractures combined with intertrochanteric fractures of the femur, often have uncertain outcomes when treated with conventional IMNs (Panteli et al., 2017). These fractures are comminuted and significantly displaced. Even with satisfactory intraoperative reduction, the forces exerted by the hip abductors, iliopsoas, and medial femoral muscles, along with the stress concentration on the internal fixation, can result in fixation failure or malunion (Bahia et al., 2019). Consistent with our findings, stress concentrations were primarily located at the fixation screw holes of the main nail, with this trend intensifying as fracture severity increased. From a micromechanical perspective, when the proximal femur undergoes axial loading, the medial wall experiences tremendous compressive forces, while the lateral wall is subjected to tension. These forces complicate the anatomical repositioning and fixation of subtrochanteric femoral fractures, frequently leading to complications, including fracture nonunion, malunion, and mechanical failure, in 7%–20% of cases (Bedi and Toan Le, 2004; Kwak et al., 2021). What, then, causes these mechanical complications?

Currently, the development of proximal femoral IMNs focus on enhancing fixation strength and counteracting compressive stress. This includes increasing the number of fixation screws, changing screw head designs, and improving the material properties of screws. Clinical outcomes have improved, but are still unsatisfactory. First, PFNA with helical blade (uniaxial) configurations have shown better torsional resistance. However, several recent studies have reported higher cut-out rate. In a study by Chapman et al. comparing IMNs in helical blade and screw groups, 7 fixation failures were reported among 126 patients, all of which occurred in the helical blade group (Chapman et al., 2018). In addition, a retrospective study by Stern et al. found that the cut-out rate was approximately five times higher in the helical blade group than in the screw group (Stern et al., 2017). Second, some researchers have suggested that two small-diameter screws (biaxial) can overcome potential weaknesses at the site of a single large-diameter screw insertion hole (Wang et al., 2000). However, this may also lead to increased stress on the cancellous bone surrounding the screw, which in turn increases the risk of cut-out (Wang et al., 2000; Brown et al., 2004). On the other hand, other authors have reported no significant differences in biomechanical testing of different screw configurations in stable fractures (Roberts et al., 2002). In cases of unstable fractures, Roberts et al. performed a biomechanical study of four different types of IMNs for treating STF (Roberts et al., 2002). The results showed increased rotation, shear, and axial translation in the group of RCN with smaller proximal diameters. Our results indicated that RCN with bi-parallel screws exhibited the highest stress, greatest displacement, and increased risk of cut-out in both types of STFs.

In addition, the dual (biaxial) screw design provides improved torsional resistance to the proximal femur. However, complications such as axial and reverse axial displacement (“Z” and “reverse Z” effects) have been observed with biaxial fixation models (Pires et al., 2011). The InterTAN system introduced a special design with double (biaxial) interlocking screws to reduce the phenomena of Z and reverse Z effects. However, the results of InterTAN are suboptimal in Seinsheimer Type V STF, especially when significant fracture displacement is present. In addition, the interlocking screw compromises the strength of the main nail at the screw hole, increasing the risk of nail fracture (Yang et al., 2024b). In Type V STF involving a coronal fracture of the greater trochanter, InterTAN fixation may result in dehiscence of the greater trochanter and proximal dislodgement of the intramedullary nail (Yalın et al., 2023). This phenomenon is known as the V-effect. It results in lateral wall damage, loss of reduction, and serious implications for proximal femoral stability (Nie et al., 2020).

The failure of internal fixation is closely related to poor fracture reduction, premature weight bearing, and improper placement of internal fixation implants. However, the primary determinant is the inability of conventional internal fixation to effectively reconstruct the tension-side trabecular system, which fails to counteract the significant tensile and partial compressive stresses produced by the bending moment of the proximal femur (Zhang et al., 2009; Yang et al., 2024b). According to Wolff’s law, the proximal femur develops a unique medial and lateral trabecular system to accommodate the compressive and tensile stresses, forming a Ward’s triangle in the central region of the proximal femur. From a biomechanical point of view, the posterior medial and lateral walls of the proximal femoral cortical bone act as fulcrums for the pressure and tension trabeculae to transmit stresses, counteracting changes in the bending moment of the proximal femur. This effectively resists inversion, rotation, and stem displacement, prevents screw cutting, and enhances the stability of proximal femoral fractures, thereby promoting fracture healing. If the integrity of these microstructures is damaged, the rate of postoperative internal fixation failure will increase by 1–2 times, and the risk of secondary surgery will increase by 6 times (Palm et al., 2007; Wu et al., 2019).

Zhang et al. proposed a set of macroscopic and microscopic triangles based on Ward’s triangle, called Zhang’s N-triangle theory (Zhang et al., 2009; Yang et al., 2024b; Zhang et al., 2024). This theory suggests that the design of internal fixation devices for the proximal femur should fully consider the need to counteract both tensile and compressive stresses. By restoring the compressive and tensile trabeculae, the physiological homeostasis of the proximal femur is restored, thereby improving the quality of fracture healing and reducing the failure rate of internal fixation. The innovation of the PFBN over conventional intramedullary nails is its double triangular structure. The first triangle, known as the mixed triangle, consists of cancellous bone, fixation screws and support screws. The second triangle, known as the metal triangle, includes the main nail, fixation screws and support screws. The double triangular structure is similar to the microscopic triangular configuration formed by the proximal femoral trabeculae under physiologic conditions.

We all know that absolute stability does not always achieve optimal fracture healing. Although torsional displacement differences between implants were minor (1.33%–5.57%), the PFBN demonstrated significantly lower von Mises stress (VMS) under torsion, with reductions of 1.02%–33.43% compared to other groups. This biomechanical advantage suggests a reduced risk of fatigue-related implant failure in long-term clinical scenarios, particularly in osteoporotic patients. In the PFBN design, the support screw locks into the main nail through its threaded end, providing firm fixation and reducing axial and torsional displacement. In contrast, the fixation screw maintains a sliding relationship with the main nail, allowing controlled micromotion at the fracture site. This locking-sliding mechanism provides functional stability, which not only prevents mechanical complications, but also enhances fracture healing by balancing stability and controlled micromotion.

Furthermore, although the differences in tangential micromotion between implants were minimal, these small variations can significantly influence implant stability and bone healing. Zhang et al. demonstrated that small tangential micromotion can reduce displacement at the fracture site, thereby helping to maintain initial fixation stability and improve fracture healing outcomes (Zhang et al., 2023). Another study comparing prosthesis designs found that reduced tangential micromotion at the bone-implant interface reduced the risk of loosening and improved long-term implant stability (Zhang et al., 2022a). Therefore, in this study, the PFBN had lower tangential micromotion values, which improved fracture healing and reduced the risk of implant-related complications.

Our results showed that the superior biomechanical properties of the PFBN under axial, bending, and torsional loads not only reduced stress at the fracture site, but also improved structural stability. For the implanted femoral head screws, the cross-design stress distribution of the fixation and support screws was superior to that of the double parallel screws, interlocking double screws, and helical blade. This stability is critical in preventing complications such as Z effect, cut-out and varus collapse, which are common with conventional IMNs. A clinical study proved our point with significantly lower postoperative pain scores and complication rates and significantly improved postoperative ambulation scores in the PFBN group compared to the PFNA group (Yang et al., 2023). In addition, by maintaining fracture alignment and load distribution during the early stages of healing, PFBN promotes the biomechanical processes necessary for osseointegration and fracture healing. Clinical reports showed that the time to fracture healing at 12 months postoperatively was shorter in the PFBN group than in the InterTAN group, and the cervical shaft angle was greater than in the InterTAN group (Jin et al., 2024).

Our study has several limitations. First, the assumption of isotropic, linear elastic materials overlooks bone heterogeneity and viscoelasticity. However, we assigned material properties to the femur model based on gray scale values, and the femur model was biomechanically validated using the GOM non-contact optical strain measurement system, which ensured the accuracy of the study. Second, the plane osteotomy model has certain limitations and is different from the actual fracture line (Zhan et al., 2024). Third, the exclusion of soft tissues (e.g., muscles, ligaments) in the current model stems from the challenges in modeling their dynamic mechanical properties and the absence of standardized biomechanical parameters under physiological loading conditions. Fourth, this study did not evaluate the PFBN’s biocompatibility or long-term clinical outcomes, necessitating future prospective studies to address these limitations.

## Conclusion

The finite element results indicate that the long PFBN demonstrates superior mechanical performance in the treatment of subtrochanteric femoral fractures under axial, bending, and torsional loads. The triangular support structure is superior to the double parallel screws, interlocking double screws, and helical blade, showing potential advantages in reducing implant-related mechanical complications.

## Data Availability

The original contributions presented in the study are included in the article/supplementary material, further inquiries can be directed to the corresponding authors.
